# Posterior-Only Approach for the Treatment of Adolescent Idiopathic Scoliosis Using Pedicle Screw Fixation: Our Experience

**DOI:** 10.7759/cureus.94514

**Published:** 2025-10-13

**Authors:** Shahzaib Riaz Baloch, Imtiaz A Hashmi, Mohammad Sohail Rafi, Syed Ata-ur-Rahman, Durdana Riaz, Raghad M Barri, Eamaan Abid

**Affiliations:** 1 Orthopedics, Ziauddin University, Karachi, PAK; 2 Orthopedics, Dr. Ziauddin Hospital, Clifton Campus, Karachi, PAK; 3 Orthopedics and Traumatology, King's College Hospital, Dubai, ARE; 4 Dentistry, Teaching Hospital Turbat, Turbat, PAK; 5 Orthopedic Surgery, Dr. Soliman Fakeeh Hospital, Riyadh, SAU; 6 Orthopedic and Spine Surgery, Ziauddin University, Karachi, PAK

**Keywords:** adolescent idiopathic scoliosis (ais), corrective spine deformity surgery, pediatric spine surgery, pedical screw, posterior spinal instrumented fusion

## Abstract

Objective: The study aims to evaluate clinicoradiological outcomes five years post-surgery, emphasizing the mid-term efficacy of posterior spinal instrumentation (PSI) and fusion using a posterior-only approach for the surgical management of adolescent idiopathic scoliosis (AIS).

Study design: This was a retrospective observational study.

Summary of background: AIS is the most prevalent spinal deformity among children. It is a three-dimensional deformity that is characterized by varying sagittal, coronal, and rotational alignment of the spine. For severe (Cobb angle 45° to 59°) and very severe (Cobb angle > 60°), PSI, along with anterior release, was traditionally the preferred method.

Methods: This study focuses on a cohort of 225 individuals who fulfilled the inclusion criteria and underwent posterior-only surgery utilizing all pedicle screw constructs for the treatment of AIS from January 2012 to December 2018. Data from the electronic records at Dr. Ziauddin Hospital, Karachi, Pakistan, were extracted to analyze functional and radiographic outcomes up to five years post surgery.

Results: The majority of our patients were females (162, 72%), with a mean age of 17.5 years for males and 14.9 years for females. The majority of AIS patients in the study were Lenke type 1 (135, 60%). In patients with neutral balance, the sagittal balance significantly improved in 204 (90.7%) at one year and 220 (97.8%) at five years. Our patients' coronal balance at one year after surgery was 216 (96%) and 221 (98.2%) at five years, as opposed to 131 (58.2%) before surgery. The mean immediate corrected angle was 8.71°, the mean one-year postoperative angle was 9.47°, and the mean five-year postoperative angle was 10.87°, compared to a mean of 72.93° preoperatively. The Oswestry Disability Index (ODI) showed significant improvement one year post operation. By five years, no patients had severe disability, but nearly all (219, 97.3%) had mild, occasional back pain. The Spine Research Society Score-22 (SRS-22) outcome measure showed improvement from a mean score of 3.69 to 4.67. The greatest improvement was seen in satisfaction and self-perceived image components.

Conclusion: Our study confirms the efficacy of a posterior-only approach using pedicle screw instrumentation in achieving stable correction of severe and very severe AIS, with minimal reported complications like surgical site infection and a few requiring revision surgery.

## Introduction

In children, adolescent idiopathic scoliosis (AIS) is the most common spinal deformity. Research indicates that the incidence ranges from 2% to 3%. A three-dimensional deformity known as AIS is typified by a vertebral rotation at the curve's apex with a curvature measuring 10° or more on a coronal X-ray, a possible modification of the sagittal profile, and lateral deviation of the spine. The most common sagittal changes, either a decrease (hypo-kyphosis) or an increase (hyper-kyphosis), are seen in the thoracic region [1].

Late childhood or adolescence is when AIS first manifests, and there is a significant chance that the curve will worsen during the pubertal growth spurt, potentially increasing the patient's morbidity. Aggressive patterns, postponed diagnosis and treatment, or a combination of the two may cause severe curve progression [2]. When the Cobb angle is greater than 45°-50° at skeletal maturity, there is a significant chance of curve advancement in adulthood [3]. For this type of deformity to significantly improve, it must be sufficiently mobilized. In order to avoid neurological and clinical complications, extensive surgery is frequently necessary. Osteotomies, apical vertebral resection, internal temporary distraction, halo traction, and anterior releases are frequently combined to get the best results [4].

Very severe idiopathic scoliosis with a Cobb angle > 80° and rigid scoliosis was previously treated with posterior spinal instrumentation (PSI) and fusion, along with a thoracotomy for anterior release, which produced a satisfactory three-dimensional correction [5]. However, anterior procedures are not the best option for patients with severe curves, as these patients frequently have cardiopulmonary limitations [6]. As a part of staged correction, some surgeons have also recommended internal distraction or preoperative traction to obtain improved correction and fusion in a shorter amount of time. On the other hand, preoperative traction is associated with a heightened risk of perioperative complications, including cranial nerve palsies, pin infections, and pin loosening [7,8]. All posterior vertebral column resections or combined anterior and posterior have also been used to correct rigid and severe scoliosis; however, this difficult technique has a number of complications [9-11]. The all-posterior approach with its dependable three-columnar fixations utilizing all pedicle screw constructs became well-liked. In actuality, the application of strong corrective forces eliminated the need for anterior release spine mobilization [12].

Notable progress has been made in the arena of spinal instrumentation over the past 30 years, particularly with regard to AIS. Harrington rods, which were originally primarily meant to apply distractive forces to the spine, gave way to pedicle screw instrumentation, which enables three-dimensional deformity correction, as the surgical (instrumentation) technique for managing scoliotic deformity. Some authors have proposed that the surgical treatment of severe AIS should switch to posterior-only fusion to avoid the deleterious effects of anterior release on pulmonary function (especially with a severe thoracic curve) [13].

The two objectives of this method are to obtain a three-dimensional correction of the scoliosis deformity and a solid intervertebral fusion. The long-term consequences and efficacy of such fixations, however, continue to raise some questions [14]. Our study's objective is to evaluate, up to five years after surgery, the clinic-radiological results of posterior spinal fusion (PSF) and PSI, via a posterior-only technique, for the surgical management of AIS.

## Materials and methods

At our facility, 381 patients underwent scoliosis correction surgery between January 2012 and December 2018. Among these, 298 had surgery limited to the posterior region using all pedicle screw constructs for AIS, and only 225 were included in the study, as some had been lost to follow-up or had incomplete medical records, resulting in not satisfying the inclusion criteria (Table 1). The distal radius ulna (DRU) classification for skeletal maturity, Risser's staging, and closure of the triradiate cartilage, along with the status of menarche, were all used to assess the maturity of the spine.

**Table 1:** Inclusion and Exclusion Criteria

S. No.	Inclusion Criteria	Exclusion Criteria
1.	Individuals with Lenke types 1, 2, 3, 4, 5, and 6 who have been diagnosed with adolescent idiopathic scoliosis (AIS).	Patients with a history of spinal surgery, regardless of the cause.
2.	Under 40 years old at the time of the procedure.	Individuals who have undergone further anterior release.
3.	Whose spine was corrected via posterior spinal fusion with just pedicle screws, rods, and/or connectors with bone grafting.	Additional specific causes of scoliosis, such as neuromuscular scoliosis, congenital scoliosis, and a number of syndromes (not including AIS)
4.	With a follow-up available for a minimum of five years	Any patient who does not meet the requirements for inclusion.
5.	Complete medical record along with pre-op, immediate post-op, and one-year and then five years post-op plain anteroposterior and lateral radiographs.	

The hospital's electronic records were the source of data collection. Radiographic measurements taken prior to surgery, immediately following surgery, and one and five years post-surgery were all documented, along with demographic information. Included were the Scoliosis Research Society-22 (SRS-22) score and the Oswestry Disability Index (ODI) score, which were recorded before surgery and one year after the procedure. The number of levels fused, the number of screws utilized, and the amount of blood loss during the procedure were all discussed. Preoperative symptoms, the Lenke curve type, and the Risser stage were noted.

Surgical steps

To make intraoperative fluoroscopy easier to use, all patients were placed prone on a radiolucent table. We used a midline posterior technique. If feasible, screws were inserted into each of the targeted vertebrae's pedicles using a freehand drilling technique. In order to minimize exposure to X-rays, we employed the "freehand" pedicle screw technique, which uses only the posterior anatomic landmarks and very limited radiologic visualization to aid with pedicle screw positioning; all cases were performed under intraoperative neurophysiological monitoring (IONM). Additionally, it does not require navigation assistance. All patients received an implant system made of titanium alloy with diameters of 4.5, 5.5, and 6.5 mm. The assembly was made using an assembly system made of 5.5 mm chrome-cobalt alloy rods.

Measurements

All radiological data originated from the picture archiving and communication system (PACS) of our hospital. Radiological parameters were measured using preoperative standing whole-spine anteroposterior and lateral radiographs, taken before surgery, on postoperative day one, and at the one-year postoperative follow-up. The following variables were measured: Cobb angles; sagittal vertical axis (SVA) (measured as the parallel distance from the plumb line falling down from the center of the C7 vertebral body to the posterosuperior edge of the S1 vertebral body in standing lateral radiographs); lumbar lordosis (LL), which is determined by the angle subtended between the upper endplate of S1 and the upper endplate of L1 in standing lateral radiographs; The measurement of thoracic kyphosis (TK) in standing lateral radiographs is the angle subtended between the lower endplate of T12 and the upper endplate of T5. Coronal balance is defined as the difference between the C7 sacral vertical line (C7-CSVL) and the C7 plumb line. The average curve correction will be calculated as follows:

$\text{Average curve correction (\%) } = \left( \frac{\text{Average pre-op coronal Cobb angle} - \text{Average post-op coronal Cobb angle}}{\text{Average pre-op coronal Cobb angle}} \right) \times 100$

In addition, the following metrics were considered: apical vertebral translation (AVT), which was determined by measuring the distance between the center of the apical vertebra and the CSVL; shoulder height difference (SHD), which was measured by calculating the difference in height between the margins (upper) of both acromioclavicular joints (positive: right higher than left); and the apical vertebral rotation (AVR), which was graded using the Nash-Moe method: grade 4, the contralateral pedicle crosses the vertebra's midline; grade 3, the contralateral pedicle (pedicle in the convex side) is in the vertebra; grade 2, the pedicle disappears; grade 1, the pedicle in the concave side (the right side); and grade 0, neutral position (no rotation) [15].

ODI score

The self-completed ODI covers 10 topics, including pain intensity, lifting, self-care techniques, walking, sitting, sexual function, standing, social life, sleep quality, and travel. Patients are going to be asked to choose the statement that best sums up their health. Each question will be scored on a range of 0 to 5, wherein 0 signifies the least amount of disability and 5 indicates the greatest degree of disability. The sex question on the ODI is optional. In order to determine the index, which has a range of 0 to 100, the responses to every question are tallied, multiplied by two, and any missing values are corrected [16].

SRS-22 score

The SRS-22 score is a valid and trustworthy tool that can be used to evaluate changes in AIS after surgery. Twenty questions in total, broken down into four sets (pain, function, self-perceived image, activity, and mental health), are included in the questionnaire. Two more questions ask about the patient's satisfaction with the treatment they received. With the exception of satisfaction, which has two items, each set contains an additional five items. Every item is ranked from worst to best, or 1 to 5. Every set has a total score between 5 and 25, with the exception of satisfaction, which has a sum between 2 and 10. The means for every dimension are used to express the results. Although it is possible to obtain a subtotal sum score of the four scales with a range of 20-100, calculating the mean is the standard procedure. The total score, after accounting for the two satisfaction questions, falls between 22 and 110 [17].

The use of the ODI and SRS-22 instruments was conducted with official permission from the copyright holders, fulfilling all necessary conditions for use.

Statistical analyses

Data were analyzed using IBM SPSS Statistics software, version 24 (IBM Corp., Armonk, NY). Continuous data (like Cobb angles and SRS-22 scores) were expressed as mean ± standard deviation and compared across pre-op, one-year, and five-year time points using paired t-tests or repeated-measures ANOVA. Categorical data (like ODI classifications and spinal balance) were presented as percentages and compared using the McNemar test. A p-value of <0.05 was considered statistically significant.

Ethical considerations

Ethics guidelines were followed in the conduct of this investigation. The privacy and confidentiality of patients were rigorously upheld during the investigation. Informed and written consent was taken from all patients and consented to by their parents/guardians. Ethical review committee approval was obtained from the review committee of Ziauddin University, Karachi, Pakistan (approval number: 8191223ARORT).

## Results

Our total sample size included 225 patients, which was female predominant (162, 72%) with a mean age of 17.5 years in males and 14.9 years in females. The mean BMI of our patients was 19.7 kg/m², with a 1.03 standard deviation. The female patients had a mean age of menarche of 12 years; out of the 162 (72%) female patients, only four (1.8%) had not achieved menarche at the time of final fusion. We had noticed that there was a mean postoperative hemoglobin (Hb) of 8.5 g/dl and SD of 1.11 in our study sample, with a mean transfusion of 1.28 pints of packed cell volume (PCV), and a total of 287 transfusions. The mean of levels fused in our 225 patients was 11, and 22.3 screws were used per patient (Table 2).

**Table 2:** Patient Demographics and Clinical Characteristics Hb: hemoglobin

Characteristic	Overall Cohort (n = 225)	Male (n = 63)	Female (n = 162)
Demographics			
Age, years	—	17.5 ± 11.4	14.9 ± 4.3
Body Measurements			
Height, cm	152.4 ± 15.09	158.2 ± 14.1	150.1 ± 14.8
Weight, kg	45.8 ± 15.15	49.5 ± 14.2	44.2 ± 15.1
BMI, kg/m²	19.7 ± 3.5	19.8 ± 3.8	19.6 ± 3.9
Surgical Details			
Pre-op Hb, g/dL	10.77 ± 1.5	11.8 ± 1.8	10.3 ± 1.2
Post-op Hb, g/dL	8.5 ± 1.2	9.1 ± 1.3	8.2 ± 1.1
Blood products transfused (PCV pints), n	1.28 ± 7.50	1.4 ± 0.6	1.2 ± 0.6
Number of levels fused	11 ± 15.3	11.5 ± 2.1	10.8 ± 1.9
Number of screws used	22.3 ± 16.5	23.5 ± 3.5	21.8 ± 3.2
Female-Specific Data			
Menarche achieved, n (%)	—	—	158 (97.5%)
Age of menarche, years	—	—	12.0 ± 1.1

ODI was used to compare preoperative ODI with one-year and five-year postoperative scores, which showed significant improvement in scores at one-year postoperative; 174 (77.3%) patients were in the mild category, and six (2.7%) had severe preoperative ODI scores, whereas 45 (20%) patients had moderate ODI scores. When comparing it at five years postoperatively, it was seen that there were no patients in the severe disability column, but almost the majority had at least some mild occasional back pain and disability, with 219 (97.3%) in the mild category, showing a significant p-value of <0.05 (Table 3).

**Table 3:** ODI of Scoliosis Patients Comparing Preoperative, One-Year, and Five-Year Postoperative Scores ODI: Oswestry Disability Index

ODI Score	Preoperative	One Year Postoperative	Five Years Postoperative
Minimal (0%-20%)	174	213	219
Moderate (21%-40%)	45	12	6
Severe (41%-600%)	6	0	0
Total	225	225	225
P-Value	0.0016		

When classifying AIS scoliosis patients using Lenke’s classification system, the majority of our patients were Lenke type 1 (135, 60%), and six (2.7%) were Lenke type 2; 56 (24.9%) patients were classified as type 3, 24 (10.7%) patients as Lenke type 6, and there were one (0.4%) and three (1.3%) patients with Lenke type 4 and type 5 curves, respectively.

Table 4 demonstrates the difference in preoperative, one-year, and five-year postoperative coronal and sagittal balance of our patients. The sagittal balance had a significant improvement in 204 (90.7%) patients at one year and 220 (97.8%) at five years, with patients showing neutral balance. One-year postoperative coronal balance of our patients was seen to be 216 (96%) and 221 (98.2%) at five years, compared to 131 (58.2%) preoperatively. 

**Table 4:** Coronal and Sagittal Balance Comparisons Preoperatively, One Year, and Five Year Postoperatively Coronal balance is defined by the C7 plumb line relative to the central sacral line: positive (right), neutral (central), or negative (left). Sagittal balance is defined by the C7 plumb line's position relative to the posterosuperior corner of S1: positive (anterior), neutral (aligned), or negative (posterior).

	Coronal Balance			Sagittal Balance		
Balance	Preoperative (n)	One Year Postoperative (n)	Five Years Postoperative (n)	Preoperative (n)	One Year Postoperative (n)	Five Years Postoperative (n)
+ (Positive)	53	6	3	93	6	1
N (Neutral)	131	216	221	33	204	220
- (Negative)	41	3	1	99	15	4
Total (n)	225	225	225	225	225	225

When comparing the magnitude of Cobb's angle, we saw that the mean immediate corrected angle was 8.71°, the one-year postoperative angle was 9.47°, and the five-year postoperative angle was 10.87° from a mean of 72.93° preoperative. 

The maturity of the spine was checked by Risser staging and the status of the triradiate cartilage, along with DRU classification, in assessing skeletal maturity. The study group showed that 178 (79.1%) patients (13 (58.7%) females and 46 (20.4%) males) had a DRU grade of radius 10/ulna 9 (R10/U9), 32 (14.2%) patients (22 (9.8%) females and 10 (4.4%) males) had a DRU of R11/U9, 13 (5.8%) patients (six (2.7%) females and seven (3.1%) males) had R9/U8, and two (0.9%) females had R8/U7.

For the Risser staging at the time of fusion, we found that 169 patients were Risser stage 5, and only 51 were stage 4, but all had closed triradiate cartilage. We had five patients who were Risser stage 5, but their triradiate cartilage was open at the time of fusion. Out of the 162 (100%) female patients, 158 (97.5%) had achieved menarche at the time of fusion, with 122 (75.3%) staged as Risser stage 5 and 36 (22.2%) as Risser stage 4. 

Our study showed improved scores in all categories of SRS-22 scores when comparing preoperative and five-year postoperative, and a statistically significant p-value, as demonstrated in Table 5.

**Table 5:** Comparison of Preoperative and Five-Year Postoperative Average Scoliosis Research Society (SRS-22) Scores P-values: *P ≤ 0.05; **P < 0.0001

Variables	Preoperative	Five-Year Postoperative	Mean Change
Pain	4.01	4.62	0.61**
Function	3.92	4.66	0.74**
Self-perceived image	2.48	4.5	2.02**
Mental health	4.10	4.48	0.38*
Satisfaction	3.88	4.89	1.01**
Average sum score	3.69	4.67	0.98

Preserving motion segments offers the significant advantage of reducing the risk of adjacent segment disease (ASD). While none of our patients developed ASD at the one-year mark, we observed this complication in six patients by the five-year follow-up; notably, none of these cases required an extension of the fusion.

In our cohort, there were no screw pullouts at one year; however, by five years, eight patients (3.5%) experienced this complication. Of these, only two (0.9%) required revision surgery, which involved laminectomy and cement augmentation of the screws.

**Figure 1:**
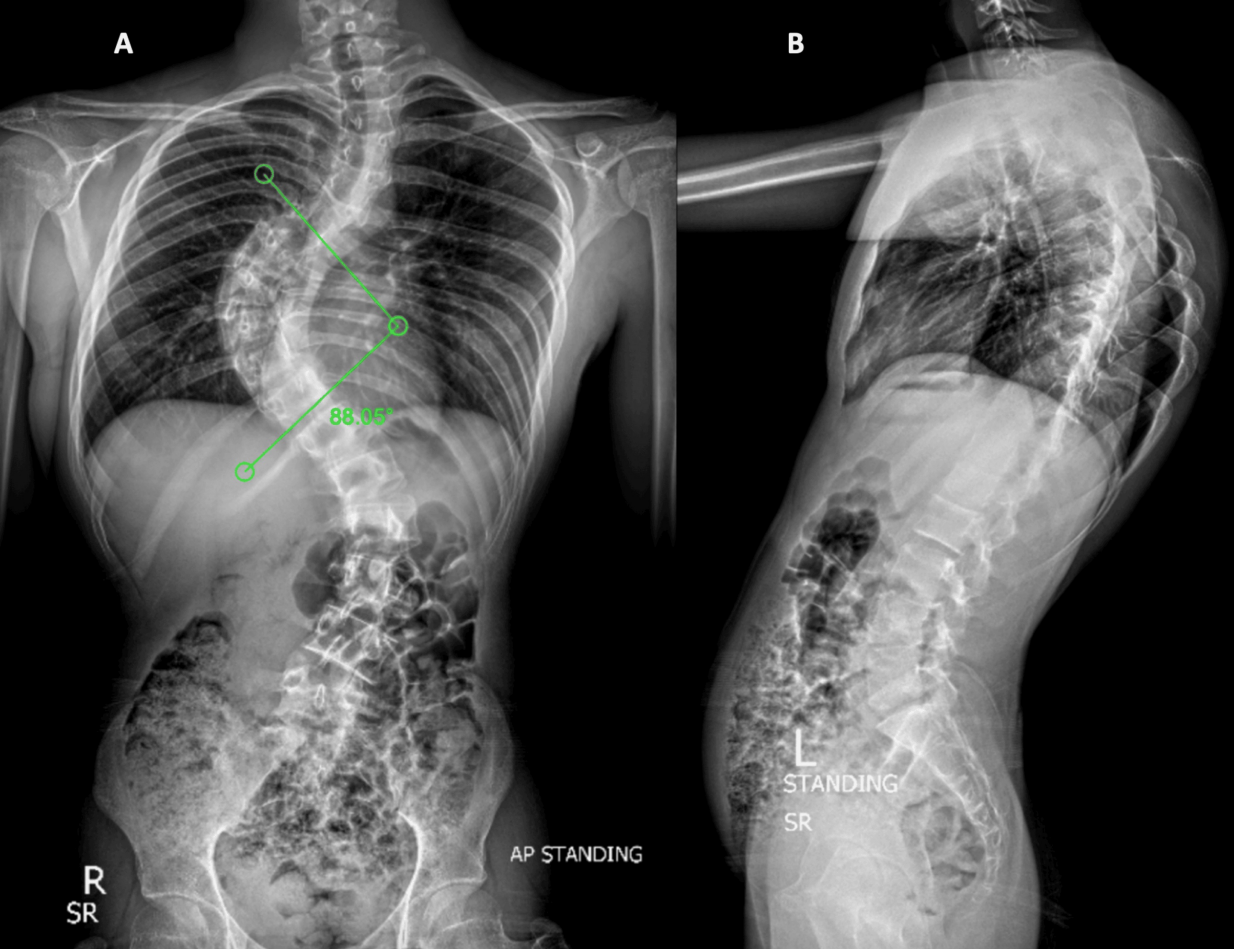
Plain Preoperative Radiographs of One of the Patients Included in the Study Posteroanterior (PA) view with a Cobb angle of 88.05°, marked as 'A', and a lateral view marked as 'B'.

Figure 1 shows the preoperative plain radiographs, posteroanterior (PA) and lateral views of the whole spine showing one of our Lenke type 3 AIS patients with Risser 5 and closed triradiate cartilage and an 88.05° Cobb’s angle of the major thoracic curve.

**Figure 2:**
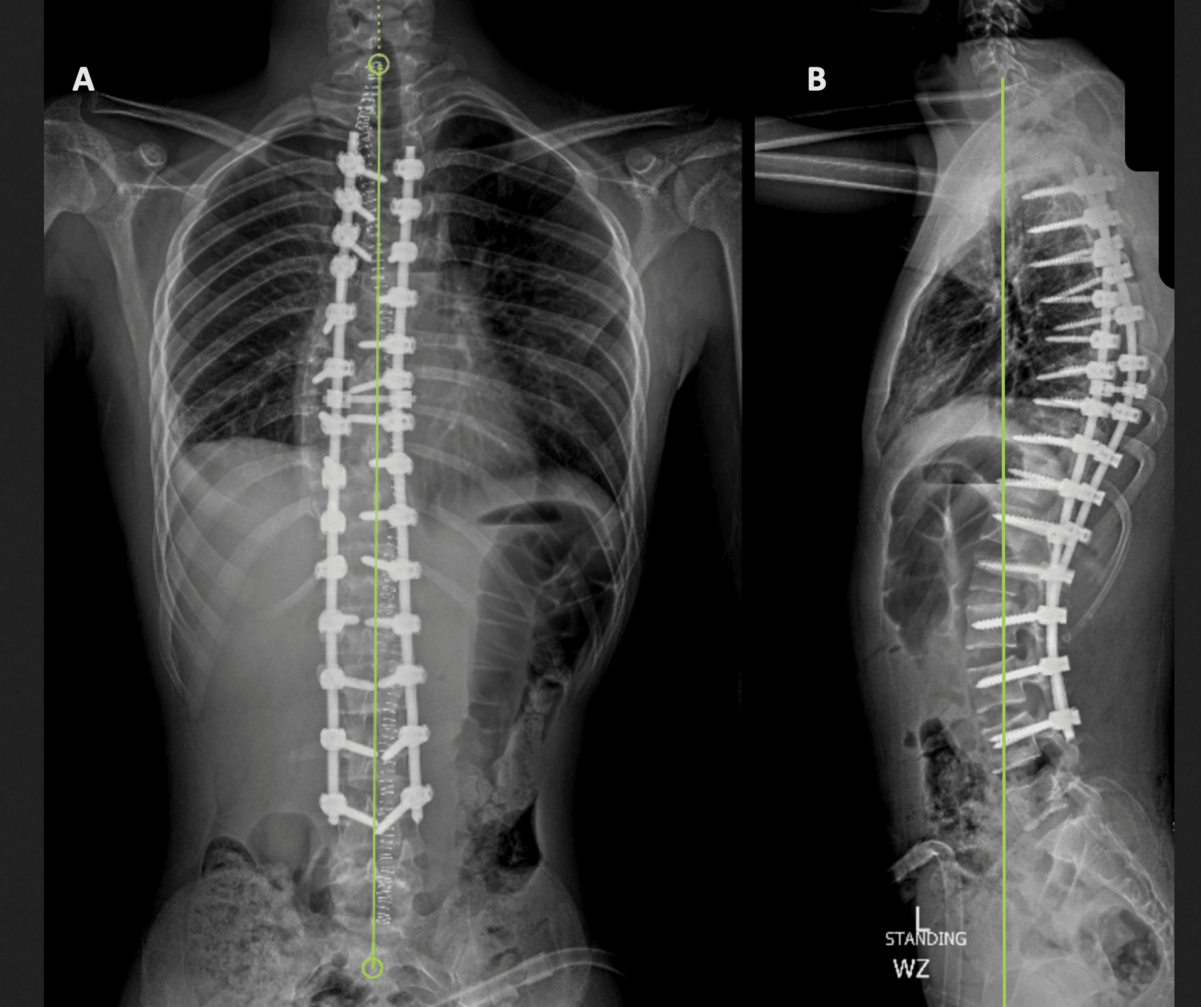
Postoperative Image of the Same Patient A posteroanterior (PA) view with a Cobb angle of 1° marked as 'A', and a lateral view marked as 'B', showing a positive sagittal balance of 39 mm.

**Figure 3:**
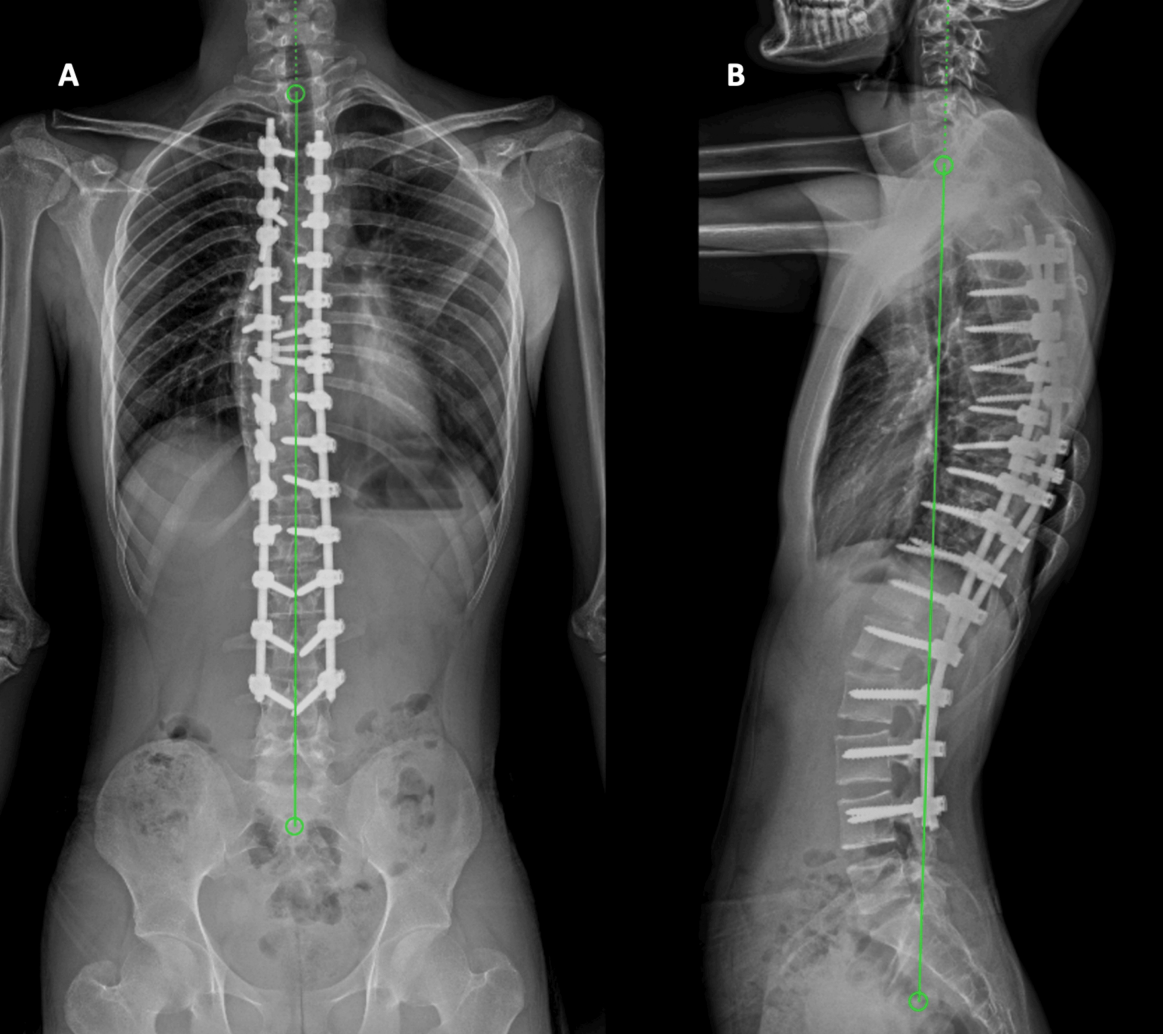
Five-Year Postoperative Plain Standing Radiograph of the Same Patient Well-maintained coronal balance with a Cobb angle of 0°, marked as 'A', and sagittal balance corrected to neutral at five years post-surgery, marked as 'B'.

Figure 2 presents plain radiographs of the PA and lateral views of the whole spine of the same patient post the surgery, demonstrating the screws in situ with no signs of loosening or screw pull-outs, with a slight positive sagittal balance. At five years postoperative, sagittal balance has corrected to neutral, and with well-maintained coronal balance, no change in Cobb’s angle can be seen even after five years post-op (Figure 3).

## Discussion

Scoliosis is a three-dimensional, complex deformity of the spine that requires a multiplanar correction. The posterior-only approach utilizing pedicle screws to anchor fixation in the strongest part of the spine is a superior technique for correction of this severe deformity [18]. This serves as an added advantage of preserving motion segments, which in turn reduces the risk of adjacent segment disease. None of our patients developed this complication up to one year postoperative, but at five years postoperative, six patients had ASD, but none required any extension of the fusion; only two patients required laminectomy plus revision with cement augmentation of screws due to screw pullout [19]. Various reports have confirmed the dominance of pedicle screws over other posterior instrumentation systems, as well as hybrid constructs, in terms of better 3D correction of the scoliotic curve and improved mechanical correction. For this reason, pedicle screws are now being widely used to treat scoliosis deformity correction [20, 21]. Our study corroborates these findings, as we did not find any screw pull-outs up to the one-year follow-up, but there were eight (3.5%) patients with screw pull-outs, and only two (0.9%) patients needed to be revised by the five-year follow-up. Furthermore, we have seen considerable correction of even very severe curves, with over 90% of the patients achieving neutral balance and maintaining it at one year postoperative.

The majority (174, 77.3%) of the patients fell into mild disability, and 45 (20%) were under the mild category preoperatively. This trend shifted towards minimal disability postoperatively, with 219 (97.3%) of our patient population landing in the minimal disability class according to the ODI and remaining with minimal disability at five years postoperative.

This study also incorporated the SRS-22 tool to evaluate clinical outcomes at one-year postoperative, which showed an improvement by an average one-point point. SRS-22 has been designed and validated to assess outcomes following surgical treatment of AIS, including relevant domains such as pain, function, mental health, and self-image. It is noteworthy that the components that scored the highest were satisfaction and self-perceived image, which is in accordance with previous studies [22, 23]. Furthermore, it is seen that the SRS-22 varies in the presence of complications, which proves its reliability as a sensitive tool in measuring functional outcomes [24].

All-screw instrumentation results in better correction than wires/hooks and hybrid instrumentation and has a lower failure rate as seen on follow-up [25]. Deeper penetration and better grip of the screws into the vertebral bodies lead to improved stability. Furthermore, they have better initial stability, which helps to achieve an effective arthrodesis maturation as compared to using other posterior instrumentation implants and even hybrid fixation. It has been stated in literature that the loss of arthrodesis correction following pedicle screw fixation is still better than when loss of correction occurs after methods of instrumented correction [2]. Moreover, screw fixation at thoracic vertebrae has improved results in terms of deformity correction, along with a decreased probability of loss of correction on follow-up. Apart from now being established as a necessary stage in the treatment of vertebral disease, it is also regarded as an essential technical necessity and considered superior to other types of instrumentation [15].

We encountered some surgical complications only, which included acute superficial surgical site infection involving six (2.6%) of our study population (only two patients), which was managed with antibiotics and dressings. Along with a total of eight patients with screw pullouts, with only two patients needing them to be revised. None of the patients had any new onset of postoperative neurological deficits. Moreover, no adverse pulmonary outcomes were noted, which is observed to be a significant con for the anterior approach [26, 27]. The SRS-22 reports an overall complication rate of 5.2% for anterior and 5.1% for posterior approaches alone, whereas a combined approach can double the risk (10%) [28, 29]. Literature states a higher association of complications with more severe curves when anterior or combined approaches are used, which in turn predisposes the patients to greater comorbidities. Hence, further reiterating the fact that the all-posterior approach has more advantages than other approaches [30].

Limitations and recommendations

A single-center retrospective design with a limited sample size is the main limitation of this study. Moreover, a multicenter study instead of a single center would give a more diverse result. Finally, follow-up is capped at five years postoperative; therefore, a longer follow-up duration would preferably offer a deeper insight as to the outcome and complications.

## Conclusions

PSF via pedicle screw-only instrumentation achieves a stable correction of severe scoliosis and results in good coronal and sagittal balance. Visceral and neurological complications following PSI for correction of scoliosis deformity are rarely reported in the literature. When a skilled spine surgeon performs the procedure, the likelihood of violating the medial pedicle wall secondary to screw misplacement appears to be low, and while results are favorable, multicenter or prospective validation would further strengthen the evidence base.
